# Evaluating the Effect of Interleukin-4 in the 3xTg Mouse Model of Alzheimer’s Disease

**DOI:** 10.3389/fnins.2020.00441

**Published:** 2020-05-14

**Authors:** Dawling A. Dionisio-Santos, Adib Behrouzi, John A. Olschowka, M. Kerry O’Banion

**Affiliations:** Department of Neuroscience, Del Monte Institute for Neuroscience, University of Rochester School of Medicine and Dentistry, Rochester, NY, United States

**Keywords:** microglia, neuroinflammation, Alzheimer’s disease, interleukin 4, tau pathology

## Abstract

Chronic neuroinflammation has long been hypothesized to be involved in Alzheimer’s Disease (AD) progression. Previous research suggests that both anti-inflammatory and inflammatory microglia ameliorate amyloid pathology, but the latter worsen tau pathology. In this study, we sought to determine whether induction of arginase-1 positive microglia with the anti-inflammatory cytokine IL-4 modulates pathology in the 3xTg mouse model of AD. Our findings indicate that a single intracranial IL-4 injection positively modulated performance of 3xTg AD mice in a Novel Object Recognition task, and locally increased the levels of arginase-1 positive myeloid cells when assessed one-week post injection. Furthermore, immunohistochemical analysis revealed decreased tau phosphorylation in IL-4 injected animals; however, we were not able to detect significant changes in tau phosphorylation utilizing Western blot. Lastly, IL-4 injection did not appear to cause significant changes in amyloid β load. In conclusion, acute intracranial IL-4 led to some positive benefits in the 3xTg mouse model of AD. Although more work remains, these results support therapeutic strategies aimed at modifying microglial activation states in neurodegenerative diseases.

## Introduction

Neuroinflammation is an established component of Alzheimer’s disease pathology with evidence that it can play both negative and beneficial roles in pathogenesis (Ghosh et al., 2013). For example, plaque-associated microglia in AD patient brain tissue express IL-1 (Griffin et al., 1989), which is thought to contribute to reactive gliosis as it stimulates the expression of other proinflammatory cytokines such as TNFα and IL-6. We previously found that sustained IL-1β expression for a period of a month or more leads to overt neuroinflammation, an increase in plaque-associated microglia and decreased Aβ plaque density (Shaftel et al., 2007; Matousek et al., 2012; Ghosh et al., 2013). While these results suggest that neuroinflammation and microglia activation are beneficial (Shaftel et al., 2008), our group and others have shown that chronic IL-1β-induced inflammation leads to an increase in pathological tau phosphorylation (Li et al., 2003; Ghosh et al., 2013).

Paradoxically, sustained IL-1β expression also induces arginase-1 expression in microglia (Cherry et al., 2015). Arginase-1 is a marker of anti-inflammatory myeloid cells, classically termed alternatively activated or M2 cells, in contrast to classically activated or M1 inflammatory cells. This M1 and M2 dichotomy is common in other models of inflammation, suggesting a balance between inflammatory and repair responses (Fenn et al., 2014). In recent years, the impression that microglia exist in two activation states has been refuted (Martinez and Gordon, 2014; Ransohoff, 2016); indeed, single cell RNA sequencing revealed that arginase-1 expression is upregulated in disease-associated microglia (Krasemann et al., 2017). These cells, which are induced by Aβ and apoptotic neurons, are highly phagocytic and also express classic inflammation markers. In agreement with these findings, arginase-1 positive microglia retain their phagocytic capabilities and are associated with plaques and the clearance of amyloid β (Cherry et al., 2015; Latta et al., 2015). Interestingly, arginase-1 overexpression in the hippocampus also led to decreased tau hyperphosphorylation in a tauopathy mouse model (Hunt et al., 2015).

One of most important cytokines that controls alternative-activation of macrophages is IL-4. In tissue macrophages, including microglia, IL-4 contributes to the diminution of pathological inflammation accompanied by the expression of anti-inflammatory molecules such as arginase-1, IL-10 and TGFβ, which aid with tissue repair (Murray et al., 2014). Given the potential beneficial role of arginase-1 positive cells in chronic neuroinflammation, we decided to determine the effect of inducing these cells with IL-4. Our past findings showed that a single intrahippocampal injection of IL-4 in APP/PS1 mice increased arginase-1-expressing microglia and decreased Aβ plaque density 5 days after injection (Cherry et al., 2015). Other groups reported similar findings with IL-13, which is another ligand of the IL-4 receptor (Kawahara et al., 2012). Interestingly, recent studies examining individuals resilient to dementia despite substantial AD pathology (Perez-Nievas et al., 2013) described increased levels of IL-13 and IL-4, among other cytokines, in the entorhinal cortex (Barroeta-Espar et al., 2019). Moreover, this change in cytokine levels was correlated with decreased gliosis and neuronal loss (Barroeta-Espar et al., 2019).

Despite these compelling observations regarding their potential utility in AD, the effect of IL-4 and other anti-inflammatory cytokines on tau phosphorylation has not been fully elucidated. We hypothesized that IL-4 induction of arginase-1 positive microglia will lead to decreased tau phosphorylation. To address this hypothesis, we performed intrahippocampal injections of recombinant murine IL-4 into 3xTg AD mice, which develop both amyloid and tau pathology with age.

## Materials and Methods

### Transgenic Mice

All animal procedures were reviewed and approved by the University Committee on Animal Resources of the University of Rochester Medical Center for compliance with federal regulations prior to the initiation of the study. Originally developed by Frank M. LaFerla and Salvatore Oddo, the 3xTg AD mice express mutated human *APP Swedish, MAPT P301L, and PSEN1 M146V* genes under the control of the Thy1.2 promoter and develop amyloid β plaque deposits and intraneuronal hyperphosphorylated tau aggregates with age (Oddo et al., 2003). We used a colony of 3xTg AD mice maintained at Rochester and bred as a homozygous line, which has been pathologically characterized (Mastrangelo and Bowers, 2008). Sixteen-month-old female 3xTg AD and non-transgenic retired breeders from a similar genetic background were used for this study. We chose to utilize female 3xTg AD mice for our study because females develop more aggressive pathology when compared to males (Carroll et al., 2010).

### Stereotactic Injections

Sixteen-month-old 3xTg AD and non-transgenic mice were anesthetized with 1.75% isoflurane, in 30% oxygen and 70% nitrogen and secured in a Kopf stereotactic apparatus using ear bars and a head holder for stereotactic injections as previously described (Ghosh et al., 2013). For bilateral hippocampal injections, stereotactic coordinates were: 2.92 mm caudal and ± 1.6 mm mediolateral relative to bregma, using a 33 GA needle lowered 1.5 mm from the brain surface over 2 minutes. For striatal injections, stereotactic coordinates were: 0.5 mm rostral, ± 2.00 mm mediolateral, using a 33 GA needle lowered 2.5 mm from the brain surface. A Micro-1 microsyringe pump controller (World Precision Instruments) was used to inject 2 μl of recombinant murine IL-4 (PeproTech) at 100 ng/μl at a constant rate over 10 min bilaterally. Control mice received similar injections with 2 μl phosphate buffered saline. Following a 5 min delay to allow diffusion, the needle was raised slowly over 2 min, and the injection was repeated on the opposite side. Over the course of the study, a total of 63 mice received bilateral injections, 43 3xTg AD (22 IL-4; 21 PBS) and 20 non-transgenic mice (10 IL-4; 10 PBS).

### Behavioral Assays

#### Novel Object Recognition (NOR)

All behavioral tasks were performed during the sleep cycle of the animals. For 3 days before behavioral testing, mice were transported from the colony room to the testing room, handled for 2 min each, and returned to the colony room to acclimate to experimenter manipulation. During the NOR habituation phase, mice were allowed to explore a 31 × 31 cm box for 10 min containing two identical objects spaced ∼15 cm apart. All objects used were ceramic doorknobs of 5–6 cm in height and ∼ 3 cm in width. Objects and chambers were washed thoroughly with 70% ethanol before each trial. Two hours after the habituation phase, the mouse was returned to the experimental cage containing the object to which it was previously exposed (familiar object; FO) as well as a novel object (NO). Placement of the NO was randomized for each mouse. Mice were allowed to explore familiar and novel objects during a 5 min test that was videotaped for subsequent analysis using AnyMaze Software. Scoring of the NOR performance was based on the time spent to explore both familiar and novel objects. The behavior of the mouse was considered explorative when the animal’s head faced the object with the neck extended and vibrissae moving. Simple proximity, passing-by or standing on the object did not count as exploration. Mice that spent less that 20 s exploring both objects were not included in the analysis. Novel object exploration was quantified as discrimination index defined by the following formula: *(Time with NO − Time with FO)/Time with NO* + *Time with FO)*.

#### Contextual Fear Conditioning (CFC)

Immediately after the NOR task was finished, mice underwent cued and contextual fear conditioning, as previously described (Matousek et al., 2010). Briefly, on conditioning day, mice were allowed to explore the conditioning context, which consisted of a Plexiglas chamber and metal floor grid. After 3 min, 15 s of white noise was presented co-terminating with a 2 s, 0.75 mA foot shock. This noise-shock pairing was repeated twice for a total of 3 shocks with a 30 s interval between shocks. Twenty-four hours later, mice were re-exposed to the conditioning chamber for 5 min each to test long-term contextual memory. Four hours later, mice were placed in a novel context consisting of a 15 cm open-topped plastic cylinder with bedding on the floor for 3 min followed by re-exposure to the white noise for 3 min, to test hippocampal-independent memory. All data were video recorded and analyzed by FreezeView Software (Coulbourn Instruments).

### Immunohistochemistry

Seven days after IL-4 injection, mice were deeply anesthetized with a mixture of ketamine (i.p 100 mg/kg) and xylazine (i.p 10 mg/kg), then perfused intracardially with 0.15 M phosphate buffered saline (PBS) containing 0.5% sodium nitrite (weight/volume) and 2 IU heparin/ml. After perfusion, brains were collected and split in half. For immunohistochemistry, one half was fixed over-night in 4% paraformaldehyde, pH 7.2 in 0.15 M PBS at 4°C. Brains were equilibrated in 30% sucrose in PBS overnight, frozen in cold isopentane and stored at −80°C. Frozen brains were then cryosectioned into 30 μm sections on a −25°C freezing stage microtome and free-floating sections were stored in a cryoprotectant solution until assayed. Immunohistochemistry using 3,3-diaminobenzidine or fluorescent immunostaining was carried out as previously described (Ghosh et al., 2013) using the following primary and secondary antibody dilutions: biotinylated 6E10 (Invitrogen) 1:2000; Iba1 (Wako) 1:3000; arginase-1 (Santa Cruz) 1:1000; P2RY12 (Invitrogen) 1:3000; biotinylated HT7 (Thermo Scientific) 1:10; pT205 (Invitrogen) 1:5000; PHF-1 (gift of Dr. Peter Davies) 1:100; biotinylated donkey anti-rabbit and anti-mouse (Vector) 1:2000; donkey anti-goat IgG Alexa 488 (Invitrogen) 1:1000; donkey anti-rabbit IgG Alexa 594 (Invitrogen) 1:1000; streptavidin Alexa 594 (Invitrogen) 1:3000. For Aβ immunostaining, sections were incubated with 70% formic acid for 3 min prior to blocking and primary antibody incubation.

### Image Acquisition and Analysis

Images were captured on a Zeiss Axioplan IIi microscope equipped with a Sensicam (Cooke Corporations), using Slidebook 6.0 software (Intelligent Imaging Innovations, Inc.). For 6E10 histological analysis, 8-Bit grayscale images from the hippocampus were captured using a 5x objective. Subiculum boundaries were defined in ImageJ^1^ and area fractions were determined using a threshold to minimize artifact. Phospho-tau and total tau stained sections were imaged using a 10x objective. CA1 boundaries were defined in ImageJ and area fractions were determined using a threshold to minimize artifact. Confocal images were obtained using an Olympus FV1000 laser scanning confocal microscope in the Confocal and Conventional Microscopy Core of the University of Rochester Medical Center Core Facility Program. All images were acquired using sequential scanning and oversaturation was prevented by using the hi-lo feature of the FV1000 software. UPLAN objectives were used to acquire the images.

### Flow Cytometry

Isolation of microglia was performed as previously described (Rivera-Escalera et al., 2019). Briefly, mice were deeply anesthetized with a mixture of ketamine (i.p. 100 mg/kg) and xylazine (i.p. 10 mg/kg), perfused intracardially with 0.15 M phosphate buffered saline (PBS) containing 0.5% sodium nitrite (weight/volume) and 2 IU heparin/ml. Brains were removed, hippocampi dissected, and hippocampal tissue was homogenized using a Dounce homogenizer. Myelin was removed by magnetic separation using myelin depletion beads and LS columns (Miltenyi Biotec). Following myelin removal, cells were washed with FACS buffer (1 × PBS containing 0.05% BSA), incubated in Fc block (1:100; BioLegend), and stained with CD11b-Alexa Fluor 488 (1:200; BD Pharmingen) and CD45-APC (1:400; BD Pharmingen). Propidium iodide (PI) was used as a viability marker. Samples were analyzed on an LSRII cytometer (BD Biosciences).

### ELISA and Western Blots

Mice were perfused and hippocampi were quickly dissected, frozen in isopentane, and stored at −80°C until further processing. Hippocampi were homogenized in Tissue Protein Extraction Reagent (T-PER; Thermo Scientific) at a concentration of 50 mg/ml with protease and phosphatase inhibitor tablets (Thermo Scientific), vortexed and sonicated. Briefly, lysates were centrifuged at 100,000 *g* for 1 h to separate monomeric and oligomeric forms of Aβ from the larger, fibrillar deposits. The supernatant was carefully collected and stored at −80°C. This was analyzed as the soluble fraction, bearing both monomeric and oligomeric forms of Aβ. The pellet, bearing insoluble, fibrillar Aβ, was extracted in guanidinium-HCl pH 6.0 (150 mg/ml) and centrifuged at 100,000 *g* for 1 h. The supernatant was stored at −80°C to be analyzed as the insoluble fraction. Levels of Aβ 1-40 and 1-42 in soluble and insoluble fraction were measured utilizing a human Aβ ELISA kit (Invitrogen). Soluble samples were diluted 1:5 in kit buffer. Insoluble samples were diluted 1:1000 in kit buffer.

For Western blot, total hippocampal lysates were diluted 1:5 and protein concentration was determined by a BCA assay (Thermo Scientific). Protein (15 μg/per lane for most blots) was electrophoresed on a Tris-HCL polyacrylamide gel and transferred to a nitrocellulose membrane (Bio-Rad) for 1 h at 4°C. After 1 h in Western blocking reagent (Roche Diagnostics), membranes were incubated overnight with primary antibodies. After rinsing, blots were incubated with peroxidase-linked secondary antibodies (Thermo Scientific), treated with the ECL substrate (Supersignal WestDura Kit) and bands were visualized using the Azure c600 imaging systems (Azure Biosystems). List of primary antibodies: HT7 (Dako) 1:1000; pT205 (Invitrogen) 1:1000; PHF1 (gift of Dr. Peter Davies) 1:1000.

### Statistics

Observers were blinded to subject treatment prior to behavioral tests and immunohistochemistry analysis. All statistical comparisons were performed using Prism 7.0 (Graphpad Software). A *p*-value ≤ 0.05 was considered significant. Shapiro-Wilk test was used to determine normality of the data. Student’s *t*-test or Mann Whitney test was employed when two group means were compared. Results where more than two group means were compared were analyzed with one-, two-, or three-way Analysis of Variance (ANOVA). A Tukey’s multiple comparison was used to establish significance between individual groups in such instances.

## Results

### IL-4 Improves Cognitive Performance in the 3xTg AD Mouse Model

**FIGURE 1:**
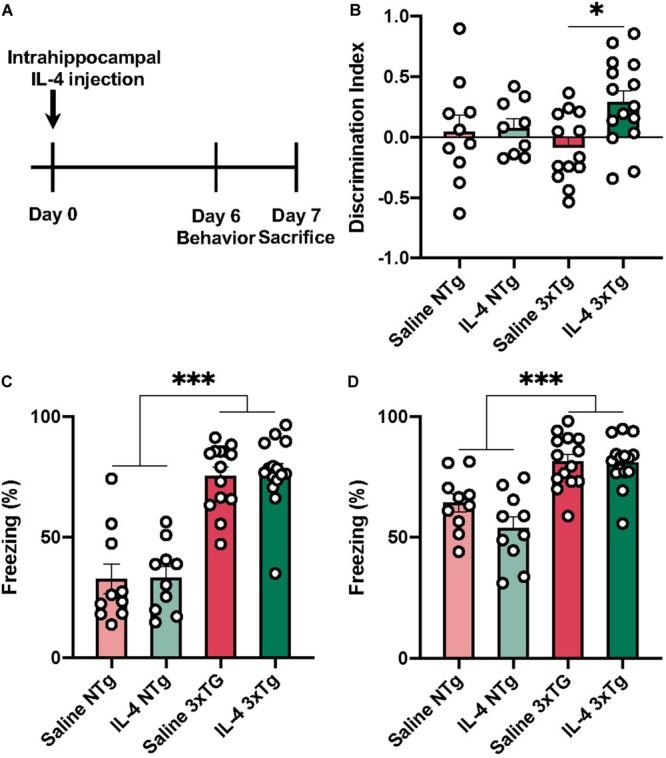
Behavioral assessment of 3xTg AD 6 days after IL-4 injection. **(A)** Experimental time-course. **(B)** IL-4 injected 3xTg AD transgenic mice have a significantly higher discrimination index when compared to saline injected transgenic mice, but not IL-4 or saline injected non-transgenic mice. 3xTg AD mice have a higher freezing response in both conditioned context **(C)** and unconditioned context with conditioned tone when compared to non-transgenic mice **(D)**. Numerical data represented as discrimination index **(B)** or freezing% **(C,D)** ± SEM; *n* = 10–15 per group. ^∗^*p* < 0.05 ^∗∗∗^*p* < 0.001 One-way ANOVA with Tukey’s *post hoc*.

In this study we sought to determine effects of the anti-inflammatory cytokine IL-4 on neuropathology and cognition in 3xTg mice. This mouse model is known for displaying deficits in spatial and contextual learning paradigms (Parachikova et al., 2010; Sterniczuk et al., 2010). To test cognitive behavior, we conducted NOR and CFC behavioral tasks 6 days after IL-4 injection (Figure 1A). One-way ANOVA revealed a significant effect of IL-4 treatment on the discrimination index in the NOR task [Figure 1B; *F* (3, 43) = 3.008, P = 0.0405]. Tukey’s *post hoc* test showed that IL-4 injected 3xTg AD mice had improved cognitive performance in NOR, as measured by a higher discrimination index when compared to saline-injected 3xTg AD animals (*P* = 0.0259). We did not find evidence of discrimination between the novel or familiar object for either group of non-transgenic mice.

We found no evidence that IL-4 affected performance in the CFC paradigm (Figures 1C,D). However, we found that both 3xTg AD groups had a significantly higher freezing response for both conditioned context (**C**) and tone (**D**) when compared to non-transgenic mice [*F* (3, 45) = 15.33 *P* < 0.0001].

### IL-4 Induces Local Expression of Arginase-1 Myeloid Cells in 3xTg AD Mice

**FIGURE 2:**
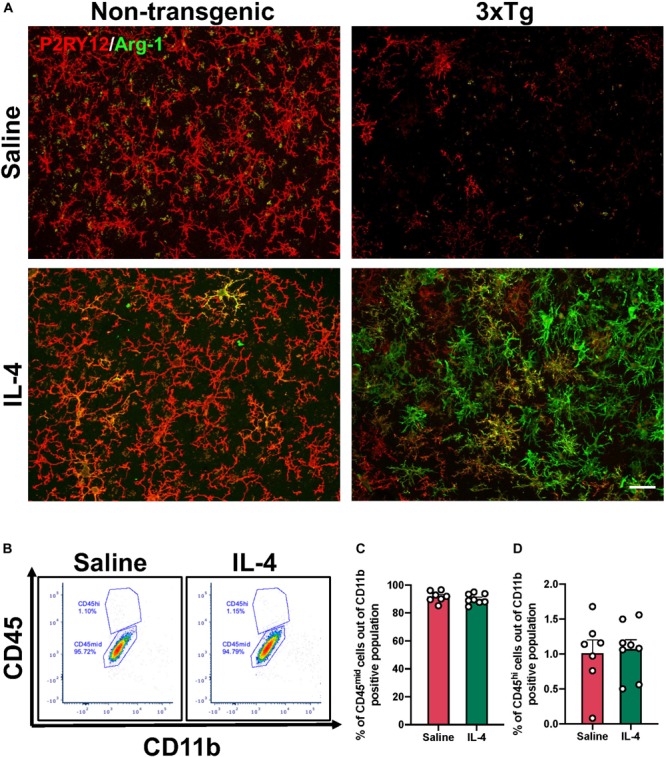
Arginase-1 expression in the subiculum after IL-4 injection. **(A)** Representative images of subiculum of 16-month-old 3xTg AD and non-transgenic mice stained with arginase-1 and P2RY12 to specifically label microglia after saline or IL-4 injection (scale bar, 25 μm). **(B)** Representative flow cytometry plots proportion of CD45^hi^ (monocytes) and CD45^mid^ (microglia) cells in the CD11b positive population isolated from the hippocampus after saline or IL-4 injection. **(C,D)** Numerical data represented as% of CD45^mid^ **(C)** and CD45^hi^ **(D)** cells in the CD11b positive population ± SEM; *n* = 7–8 per group.

IL-4 is a potent type 2 cytokine that can drive tissue macrophages to acquire an alternatively activated phenotype. Our immunofluorescence results showed low baseline expression of arginase-1 around areas of amyloid accumulation that was dramatically increased by acute IL4 injection (Figure 2).

We sought to determine whether these arginase-1 positive cells were microglia or infiltrating peripheral cells by performing co-immunostaining with the microglia specific marker P2RY12. Qualitative observations revealed that, some of the arginase-1 positive cells, but not all, co-localized with P2RY12 (Figure 2A). However, P2RY12 staining was low in the subiculum of 3xTg AD mice at baseline (Figure 2A). To determine whether the increase in arginase-1 positive cells was due to infiltrating monocytes, we carried out flow cytometry. We found that IL-4 did not increase the proportion of CD45^hi^ monocytes in the hippocampus of 3xTg AD (Figures 2B–D).

Interestingly, IL-4 dependent arginase-1 expression was greater in 3xTg compared to non-transgenic mice (Figure 2A). To test the possibility that this was due to microglial sensitization in the vicinity of Aβ plaques, we also carried out IL-4 injections in the striatum, which does not accumulate extracellular Aβ plaques in the 3xTg AD mouse and quantified the number of arginase-1 positive cells (Supplementary Figure 1). Three-way ANOVA revealed that treatment [*F* (1, 24) = 79.90 *P* < 0.0001] and genotype [*F* (1, 24) = 81.56 *P* < 0.0001] are the largest sources of variation in the data. Tukey’s *post hoc* revealed that IL-4 injection significantly increased the number of arginase-1 positive cells in the striatum and hippocampus of 3xTg AD when compared to the number of arginase-1 positive cells in the same regions in the non-transgenic (*P* < 0.0001) and all groups with saline injection (*P* < 0.0001).

### Effect of Acute IL-4 Injection on 3xTg AD Neuropathology

**FIGURE 3:**
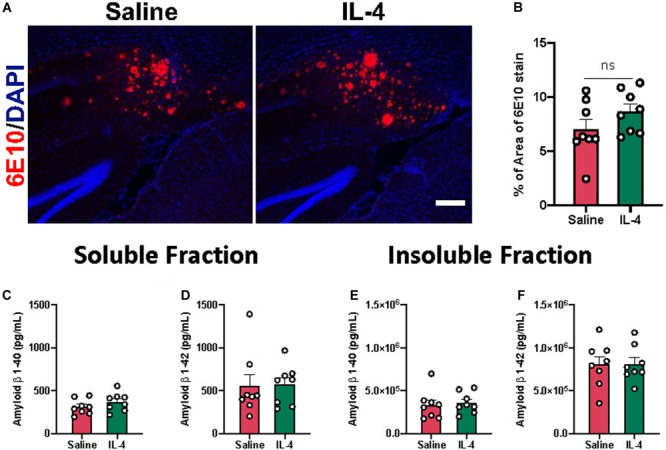
Effect of IL-4 on amyloid β plaque load in the subiculum of 3xTg AD. **(A)** Representative images of amyloid β plaques with 6E10 immunostaining in the subiculum of 16-month-old female 3xTg AD mice after saline or IL-4 injection (scale bar, 50 μm). **(B)** Percent area covered by 6E10 stain determined after threshold utilizing Image(J) software. **(C–F)** Aβ 1-40 and 1-42 levels in soluble and insoluble fraction in the hippocampus of 3xTg AD mice. Numerical data are represented as percent area **(B)** or pg/ml **(C–F)** of Aβ ± SEM; *n* = 8 per group. N.S. = not significant. Student’s *t* test.

Previously, our group reported that acute IL-4 injection results in decreased Aβ plaque area in the APP/PS1 mice (Cherry et al., 2015). However, acute IL-4 injection did not reduce 6E10 positive Aβ plaque area in the subiculum of 3xTg AD mice by immunohistochemistry (Figures 3A,B). Consistent with this observation, we did not detect an effect of IL-4 on Aβ 1-40 or 1-42 levels in soluble and insoluble fractions isolated from mouse hippocampus (Figures 3C–F).

**FIGURE 4:**
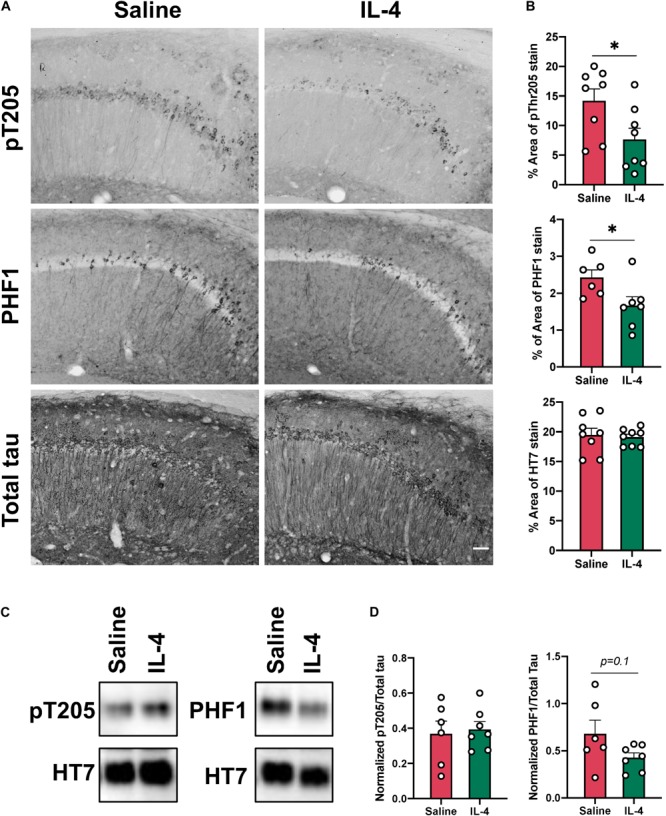
Intrahippocampal injection of IL-4 significantly decreases tau phosphorylation without changing total tau levels. **(A)** Representative images of phosphorylated tau pT205, PHF1 and total tau HT7 immunostaining in the CA1 of 16-month-old female 3xTg AD mice after saline or IL-4 injection (scale bar, 50 μm). **(B)** Percent of area covered by tau stain determined after threshold utilizing Image(J) software. **(C)** Representative immunoblots of phospho-tau epitopes in hippocampal lysates from 3xTg AD mice. **(D)** Image(J) software was used to determine band intensity and phospho-tau epitopes were normalized with total tau. Numerical data are represented as% of area mean **(B)** or normalized phospho-epitope levels **(D)** ± SEM; **(A)** *n* = 8 per group. **(C)** *n* = 6–7 per group. ^∗^*p* < 0.05, ^∗∗^*p* < 0.01. Student’s *t* test.

Given that sustained neuroinflammation in the hippocampus of 3xTg AD mice results in increased tau phosphorylation (Ghosh et al., 2013), we hypothesized that acute IL-4 injection and induction of arginase-1 positive cells could decrease tau pathology in the 3xTg AD mouse. We found evidence of a significant reduction in tau phospho-Threonine 205 (*P* = 0.043) and PHF1 (*P* = 0.0032) by immunohistochemistry one week after IL-4 administration in sections close to the injection site, which was not associated with a decrease in total tau stain (Figures 4A,B). Despite this clear change observed with stained tissue sections, we were not able to detect significant changes in phospho-Threonine 205 using Western blot of hippocampal lysates obtained from tissue collected around the injection site (Figures 4C,D). However, there was a trend toward decrease in the PHF1 epitope after IL-4 injection (*P* = 0.1), which identifies the late-stage neurofibrillary tangle (NFT) phospho-tau sites Ser396 and Ser404.

## Discussion

In this study we sought to determine the effect of the anti-inflammatory cytokine IL-4 on cognition, Aβ plaque load and tau phosphorylation in the 3xTg mouse model of AD. Previously, we demonstrated that sustained expression of the proinflammatory cytokine IL-1β by different paradigms leads to reduction of Aβ plaque pathology (Matousek et al., 2012; Ghosh et al., 2013; Rivera-Escalera et al., 2014; Cherry et al., 2015). However, chronic proinflammatory signaling can drive activation of tau kinases and led to an increase in pathological tau phosphorylation (Li et al., 2003; Ghosh et al., 2013). This is consistent with the commonly cited idea that pathological neuroinflammation is a contributing factor to the progression of disease and cognitive decline (Dionisio-Santos et al., 2019). On the other hand, sustained IL-1β also drives expression of arginase-1, a marker of anti-inflammatory myeloid cells, in microglia (Cherry et al., 2015). We previously reported that acute induction of these cells after intrahippocampal injections with IL-4 was associated with decreased Aβ plaque load (Cherry et al., 2015). T lymphocyte derived IL-4 has also been shown to modulate meningeal immunity and plays a role in preserving learning and memory (Derecki et al., 2010). Modulation of peripheral T lymphocytes with agents such as glatiramer acetate could provide a potential avenue to increase IL-4 signaling in the CNS of AD patients (Dionisio-Santos et al., 2019).

Our findings revealed that acute IL-4 injection improves performance of 3xTg AD mice in a novel object recognition task. Independent of pathology, IL-4 knockout mice perform poorly in paradigms such as the Morris Water Maze, suggesting that IL-4 plays a role in learning and memory (Derecki et al., 2010). Interestingly, non-transgenic controls did not show discrimination toward the familiar or novel object. One caveat with our behavior experiments is that we performed them during the animal’s sleep-cycle; thus, activity levels among the transgenic and non-transgenic lines might affect engagement in exploratory behavior. We found that 3xTg AD mice showed greater freezing behavior in both the context and non-context phases of the trial when compared to non-transgenic mice regardless of IL-4 injection, indicating a basal difference in performance. Reports in the literature describe increased anxiety behavior in this transgenic line, which could be responsible for the observed differences (Sterniczuk et al., 2010; Hebda-Bauer et al., 2013). One possible mechanism for such behavioral differences between 3xTg AD and non-transgenic mice could be increased brain proinflammatory cytokines, which have been reported in this AD mouse model (Janelsins et al., 2005; Kitazawa et al., 2011).

These changes in behavior were correlated with a local increase of arginase-1 positive cells in the subiculum and CA1 region of 3xTg AD hippocampus in sections near the IL-4 injection site with virtually no positive cells present in the saline injected brains. Many of these arginase-1 positive cells did not express the microglia specific marker P2RY12. However, expression of this marker is decreased near Aβ plaques, consistent with previous observations that microglial P2RY12 mRNA expression is decreased in APP/PS1 mice (Krasemann et al., 2017). Therefore, we cannot exclude the possibility that these cells are microglia. Of note, we did not find an increase in the proportion of peripheral monocytes, defined with flow cytometry as CD11b^+^, CD45^hi^ cells, in the hippocampus of 3xTg AD mice after IL-4 injection. Interestingly, IL-4 dependent induction of arginase-1 was more dramatic in 3xTg AD mice than in non-transgenic mice. Our results suggest that this was not due plaque proximity, but rather an increased sensitivity to IL-4 in 3xTg AD mice. Even though the 3xTg AD mouse does not develop extracellular plaque pathology in the striatum, this area is likely exposed to soluble Aβ oligomers, which promote neuroinflammation. In addition, 3xTg AD mice have increased brain levels of soluble cytokines, such as TNFα and CCL2 (Janelsins et al., 2005), which sensitize microglial responses. Consistent with this idea, inflammatory insults such as peripheral LPS injection and spinal cord injury induce expression of the IL-4Rα in microglia (Fenn et al., 2012, 2014).

In contrast with studies in other amyloidogenic mouse models (Kawahara et al., 2012; Cherry et al., 2015), we observed that Aβ plaque load and levels of soluble and insoluble Aβ peptides were unchanged in 3xTg mice after acute injection of IL-4. We elected to inject IL-4 at 16 months to observe appreciable tau hyperphosphorylation, which is more apparent after 15 months of age in these mice (Mastrangelo and Bowers, 2008). At 16 months of age microglia might be subject to senescent changes that reduce their ability to remove proteinaceous deposits (Hickman et al., 2008; Streit et al., 2009) when compared to other amyloidogenic mouse models, which are typically evaluated at a younger age due to the earlier onset of pathology.

On the other hand, we found some evidence of reduction in pathogenic phosphorylated tau as seen by decreased immuno-positive staining of phospho-Threonine 205 and PHF-1, markers of early and late tau pathology, respectively (Augustinack et al., 2002; Regalado-Reyes et al., 2019). These results agree with findings from Daniel C. Lee’s group showing that arginase-1 overexpression decreased tau pathology in the rTg4510 tau transgenic mouse (Hunt et al., 2015). Despite these changes in tissue staining, we were unable to detect significant phospho-epitope changes by Western immunoblotting. One possible explanation is that our microdissection was not precise enough to capture the area surrounding the injection site, where arginase-1 expression is the highest. Future studies utilizing chronic IL-4 overexpression could be evaluated; however, increased mortality has been reported in APP/PS1 transgenic mice after AAV-IL-4 intracranial injection (Latta et al., 2015).

In conclusion, we confirmed that IL-4 provides a strong stimulus for microglia in 3xTg AD mice to express arginase-1. The presence of these arginase-1 positive cells in the CA1 and subiculum was correlated with improvement in recognition memory. Our immunohistochemical analyses indicated decreased tau phosphorylation at epitopes phospho-Threonine 205 and PHF1, which agrees with studies evaluating the effect of chronic arginase-1 over-expression on tau phosphorylation (Hunt et al., 2015). While we were not able to observe changes in pathology in all of our analyses, additional evidence suggests that IL-4 might reduce gliosis and neurodegeneration in humans resilient to AD. In the future, it will be important to evaluate whether chronic induction of IL-4 signaling in the brain has potential benefits in AD.

## Data Availability Statement

The raw data supporting the conclusions of this article will be made available by the authors, without undue reservation, to any qualified researcher.

## Ethics Statement

The animal study was reviewed and approved by University Committee on Animal Resources of the University of Rochester Medical Center.

## Author Contributions

DD-S and MO’B designed the research. DD-S performed research, analyzed data, and wrote the manuscript. AB made the initial observation of increased arginase-1 expression in 3xTg mice. MO’B and JO provided input throughout the process. All authors reviewed and approved the manuscript.

## Conflict of Interest

The authors declare that the research was conducted in the absence of any commercial or financial relationships that could be construed as a potential conflict of interest.

## Footnotes

1 http://rsb.info.nih.gov/ij/
