# Supplementary material for: Evaluating the Effect of Interleukin-4 in the 3xTg Mouse Model of Alzheimer’s Disease
Source: Front Neurosci. 2020 May 14;14:441. doi: 10.3389/fnins.2020.00441 (PMC7247853; doi:10.3389/fnins.2020.00441)
Supplement: Supplementary file 1 [file Data_Sheet_1.docx]

Supplementary Material

**Supplementary Figure 1.** **Comparing IL-4 induced arginase-1 expression between 3xTg AD and non-transgenic mice.** (A) Representative images of the hippocampus and striatum of 3xTg AD and non-transgenic stained with arginase-1 after saline or murine IL-4 injection (scale bar, 25 μm). (B) Number of cells per field of view was counted in images at the injection site. Numerical data represented as average cell density ± SEM; n=4 per group. **** p<0.0001. Three-way ANOVA with Tukey’s *post hoc*.
